# Topical Application of Estrogen Nanocapsules on Skin Incision Improves Fracture Healing in Osteoporotic Rats

**DOI:** 10.1055/s-0044-1800944

**Published:** 2025-06-14

**Authors:** Dalton Berri, Elcio Machinski, Conrado Auer Trentini, Paulo Vitor Farago, Adriana Yuriko Koga, Leandro Cavalcante Lipinski

**Affiliations:** 1 Department of Medicine, Universidade Estadual de Ponta Grossa, Ponta Grossa, PR, Brazil; 2 Department of Pharmaceutical Sciences. Universidade Estadual de Ponta Grossa, Ponta Grossa, PR, Brazil

**Keywords:** estrogens, fracture healing, nanotechnology, osteoporosis, consolidação da fratura, estrogênios, nanotecnologia, osteoporose

## Abstract

**Objective** The challenge of consolidating osteoporotic fractures, particularly exacerbated by postmenopausal estrogen deficiency, underscores the urgent need for effective interventions. This study aims to evaluate the impact of locally administered estrogen via nanocapsules on the consolidation of osteoporotic fractures in ovariectomized rats, while also assessing the systemic effects of this hormone, using the uterus as a sentinel organ.

**Methods** Forty-five animals underwent standardized femoral fractures and were divided into three groups: G1 (control), G2 (conventional estrogen), and G3 (estrogen nanocapsules). The estrogen was applied topically to the skin incision region (trichotomized area). Fracture healing was assessed at 15- and 30-days postfracture through radiographic and histological analyses, with uterine histology conducted to evaluate systemic effects.

**Results** In terms of radiographic analysis of callus formation, G3 (8.75 ± 0.77 mm) exhibited significantly higher callus formation than the control group (7.18 ± 0.4 mm) at day 15, with histological analysis revealing increased callus formation in G3 at day 30, indicating an accelerated healing process. Furthermore, uterine histological analysis at day 30 showed a reduction in endometrial thickness in G3 (510,073 ± 54,705.11 μm) compared with G2 (623,729 ± 101,592 μm).

**Conclusion** These findings suggest that topical estrogen nanocapsules may enhance callus formation in the treatment of osteoporotic femoral fractures in rats, potentially with fewer systemic effects.

## Resumo

**Objetivo** O desafio de consolidação de fraturas osteoporóticas, particularmente exacerbadas pela deficiência de estrogênio pós-menopausa, ressalta a necessidade urgente de intervenções eficazes. Este estudo tem como objetivo avaliar o impacto do estrogênio administrado localmente via nanocápsulas na consolidação de fraturas osteoporóticas em ratas ovariectomizadas e analisar os efeitos sistêmicos deste hormônio usando o útero como órgão sentinela.

**Métodos** Quarenta e cinco animais foram submetidos a fraturas femorais padronizadas e divididos em três grupos: G1 (controle), G2 (estrogênio convencional) e G3 (nanocápsulas de estrogênio). O estrogênio foi aplicado topicamente na região da incisão da pele (área tricotomizada). A cicatrização da fratura foi avaliada 15 e 30 dias após a fratura por meio de análises radiográficas e histológicas. A histologia uterina analisou os efeitos sistêmicos.

**Resultados** Na análise radiográfica dos calos ósseos, G3 (8,75 ± 0,77 mm) exibiu formação de calo significativamente maior do que o grupo controle (7,18 ± 0,4 mm) no dia 15 e a análise histológica revelou aumento da formação de calo em G3 no dia 30, indicando um processo de cicatrização acelerado. Além disso, a análise histológica uterina no dia 30 mostrou uma redução na espessura endometrial em G3 (510.073 ± 54.705,11 μm) em comparação a G2 (623.729 ± 101.592 μm).

**Conclusão** Estes achados sugerem que nanocápsulas tópicas de estrogênio podem aumentar a formação de calos no tratamento de fraturas femorais osteoporóticas em ratas, potencialmente com menos efeitos sistêmicos.

## Introduction

Osteoporotic fractures represent a significant public health issue, with their incidence increasing each year. 1 2 3 Most treatment strategies focus on preventing these injuries by increasing bone mass, but there is less emphasis on the consolidation process of osteoporotic bone. 4 Additionally, the consolidation of osteoporotic fractures presents a significant challenge for orthopedic surgeons. 5

Estrogen deficiency, particularly postmenopausal, is a major risk factor for osteoporosis. This hormone has both anabolic and anticatabolic effects, influencing osteoblasts and osteoclasts in the bone remodeling process. 6 7 8 Moreover, it is crucial for the regulation and formation of cartilaginous tissue, affecting both growth cartilage and joint surfaces. 9

While the role of estrogen in bone metabolism and its protective effect on bone mineral density are well-known, 10 its impact on fracture consolidation remains unclear. Few studies have specifically evaluated the role of this hormone in the healing process of postmenopausal osteoporotic fractures. 11 12 13 The reduction of estrogen levels during menopause directly contributes to an imbalance in bone neoformation that can adversely affect the bone consolidation process in osteoporotic patients. 14 15 To mitigate these effects, topical application is advantageous, as it avoids first-pass hepatic metabolism, thereby reducing the necessary concentration and minimizing side effects. 16

Advances in nanotechnology allow for the manipulation of particles to create drug transport vehicles that safely target specific organs, improving transport effectiveness. 17 Nanomaterials have unique structures with adjustable size, shape, and surface properties that significantly impact cellular absorption. 18 In biological systems, smaller particles can be ideal for the cellular absorption of active compounds. 19 20 Nanosystems can be beneficial in drug delivery by improving the bioavailability of poorly soluble actives, reducing side effects, releasing the drug in a controlled manner, and allowing administration at lower doses. 21

In this study, we evaluated the role of topical estrogen applied to the skin incision, administered both conventionally and through nanocapsules, radiographically and histologically, in the femoral fracture consolidation process in osteoporotic rats, while also assessing the systemic effects of this hormone, with the uterus as a sentinel organ.

## Materials and Methods

### Development of Pharmaceuticals

The drugs were developed at the Laboratory of Drug Production and Development. Both the nanocapsules and conventional formulation had a concentration of 0.06% of 17-β estradiol.

### Obtaining Polymeric Nanocapsules Containing 17-β Estradiol

Nanocapsule suspensions were obtained using PCL (100 mg) dissolved in acetone (30 ml) in the presence of Span 80 (Croda International plc, Snaith, UK) at 0.077 g, 17-β estradiol (50 mg), and medium-chain triglycerides (0.33 g). The solution was stirred for 10 minutes. The aqueous phase was prepared using Tween 80 (Croda International plc) at 0.077 g, and distilled water (53 mL). Then, the organic phase was slowly added to the aqueous phase under constant magnetic stirring at 40°C. The resulting nanoemulsion was stirred for 10 minutes. Next, the organic solvent was removed by evaporation under reduced pressure at 40°C, resulting in a concentrated sample (10 mL).

### Field Emission Gun Scanning Electron Microscopy (FEG-SEM)

The morphological and surface evaluation of the nanoparticle and conventional form was performed using a Mira 3 field emission gun scanning electron microscope (TESCAN, Brno, Czech Republic). The samples were metallized with gold using an IC-50 Ion Coater (SHIMADZU, Kyoto, Japan). Electron micrographs were obtained using an acceleration voltage of 15 kV and specific software (Electron Optical Design, Brno, Czech Republic).

### Dynamic Light Scattering and Laser Doppler Microelectrophoresis

The particle size and zeta potential of the nanoparticles (E2, PCLN, and ZnON) were determined using a Zetasizer Nano series ZS90 instrument (Malvern Instruments, Worcestershire, UK) after sample preparation (1:500 V/V) in ultrapure water. The analyses were performed in triplicate.

### Animal Model

The research was approved by the Ethics Committee on Animal Use (CEUA) under process number 0122368/2019. All applicable institutional and national guidelines for the care and use of animals were followed.

There were 45 female Wistar rats divided into 3 groups. Group 1 (G1) consisted of 15 rats in the control group, group 2 (G2) consisted of 15 rats treated with estrogen conventionally formulated at a concentration of 0.06% of 17 β-estradiol, and group 3 (G3) consisted of 15 rats treated with the same concentration of 17 β-estradiol but formulated through nanocapsules.

### Anesthesic Technique

For the ovariectomy and fracture production procedures, the animals were anesthetized with xylazine (10 mg/kg) and ketamine (90 mg/kg). Postsurgery, they received a single dose of fentanyl citrate (0.05 mg/kg) for pain management, followed by dipyrone (200 mg/kg) administered every 6 hours for the first 7 days. Antiinflammatory drugs were not used to avoid potential interference with bone consolidation evaluation. Postoperative pain was monitored by assessing food and water consumption, as well as observing behavioral changes.

### Fracture Production

After 40 weeks of ovariectomy, the rats were anesthetized using the same anesthetic technique and underwent a right femur fracture. Trichotomy and antisepsis were performed with topical povidone-iodine (PVPI). A 2 cm incision was made in the lateral thigh in the right hind limb and extended to the knee, dissection was performed by planes, and the patella was retracted to expose the lateral condyles. A 1 mm diameter A Kirschner wire was inserted through the condyles to the greater trochanter and removed on day 15. After intramedullary fixation of the femur, the lateral vastus muscle was retracted, exposing the bone diaphysis. A transverse fracture was made in the femoral diaphysis using a 5 mm osteotome. After the procedure, the muscle fascia was sutured with absorbable polygalactin 2 to 0 suture and the skin with 3 to 0 mononylon. Immediate postoperative radiographs were taken to confirm the fracture.

### Estrogen Application

Groups 2 and 3 received daily application of topical estrogen according to their respective groups (paste or nanocapsules at a concentration of 0.06%) for 14 days, around the operative wound (already trichotomized area).

After drug application, each group was subdivided into 15 days postfracture and 30 days postfracture for euthanasia. Afterwards, femurs were removed and cleaned for radiographic and histological analysis.

### Radiographic Evaluation

A Lotus 630HF device was used to obtain radiographs of both femurs. The VXvue 1.0.2.6pi (Viewoks Co. Ltd., Anyang, South Korea) software was used to measure the bone callus at its largest diameter and the femoral isthmus of the contralateral femur. Absolute measurement of the bone callus and the ratio between the callus and the contralateral isthmus were evaluated.

### Histological Analysis

Bones, cleaned of muscle tissue, were stored in 10% formaldehyde and subsequently decalcified in a solution of 10% ethylenediaminetetraacetic acid (EDTA) changed weekly for 2 months. The fractured segment was embedded in paraffin, longitudinally cut to a thickness of 5 µm, and stained with hematoxylin-eosin (HE). After analysis and selection, histological sections were photographed with an Olympus DP72 (Evident Corp., Shinjuku-ku, Tokyo, Japan) microscope using the cellSens Standard (Evident Corp.) software. Qualitative and quantitative analyses of the samples were performed. The numerical scale proposed by Huo et al., 22 according to the consolidation stage observed in each slide, was applied.

### Uterine Tissue

After euthanasia, all uteri were collected and fixed in 10% formalin. Histological processing was then performed, and the sample was sectioned using a microtome with a thickness of 3 µm and stained with hematoxylin and eosin (HE). Histological sections were photographed using an Olympus AX70 (Evident Corp.) microscope with 20x magnification, using the T capture program. The thickness of the perimetrium, myometrium, and endometrium layers was measured using the ImageJ software after standardization of the known distance. The mean and standard deviation were calculated for subsequent statistical analysis.

### Statistical Analysis

Statistical evaluation was performed using the Statistical Package Social Sciences (SPSS, IBM Corp., Armonk, NY, USA) version 20.0, with analysis of variance (ANOVA) for multiple comparisons, followed by Tukey's test, with a confidence interval (CI) of 95% ( *p* ≤ 0.05).

## Results

### Nanocapsule Production Process

The evaluation of the size of estrogen nanoparticles showed a mean value of 191.96 ± 10.37 nm. Scanning electron microscopy (SEM) characterization is a technique that makes possible to evaluate the influence of synthesis conditions on the morphology of nanoparticles. The results obtained for estrogen in conventional form showed large and irregular parts. The SEM of estradiol nanoparticles showed a spherical shape with a homogeneous surface.

### Drug Concentration Determination and Encapsulation Efficiency

The determination of the drug concentration incorporated into the nanocapsules, and the encapsulation efficiency was performed in triplicate using the previously validated method. The nanocapsules obtained by the precipitation method of the preformed polymer showed yields greater than 99%.

### Radiographic Evaluation

At the 15-day assessment, all animals already showed signs of bone callus formation. After 30 days, all fractures were consolidated in their radiological aspect. The results for the width of the bone callus are substantiated in Table 1 .

**Table 1:** Mean and standard deviation of the width of the bone callus and the ratio between the width of the callus and the width of the contralateral isthmus at 15 and 30-days postfracture (in mm)

	Day 15		Day 30	
	Width	Ratio	Width	Ratio
**G1**	7.18 ± 0.4 ^a^	2.04 ± 0.19 ^a^	8.3 ± 0.97 ^a^	2.4 ± 0.36 ^a^
**G2**	8.37 ± 1.4 ^a,b^	2.43 ± 0.47 ^a,b^	8.51 ± 0.9 ^a^	2.49 ± 0.2 ^a^
**G3**	8.75 ± 0.77 ^b^	2.54 ± 0.22 ^b^	8.53 ± 1.0 ^a^	2.56 ± 0.27 ^a^

**Abbreviations:** G1, group 1; G2, group 2; G3, group 3. **Notes:** Results are displayed in median ± standard deviation. Different letters in the columns denote significant difference *p* < 0.05.

**Fig. 1:**
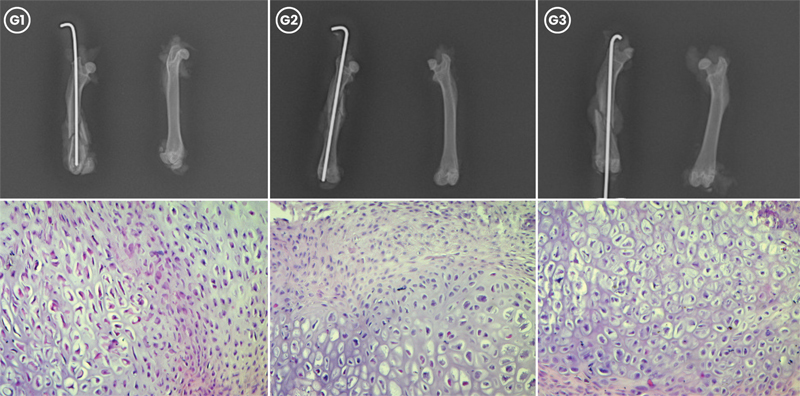
Radiographic and histological evaluation of femoral fracture healing in G1, G2, and G3 at 15 days postfracture. (Top) Radiographs showing callus formation in G1, G2, and G3. (Bottom) Histological sections of callus formation in each group, stained with hematoxylin and eosin (magnification 20x).

In the evaluation conducted at 15 days post-fracture, animals in G3 exhibited a larger bone callus than those in G1 ( *p* < 0.05), while animals in G2 showed a statistically equal callus compared with G1 and G3, as shown in Fig. 1 .

**Fig. 2:**
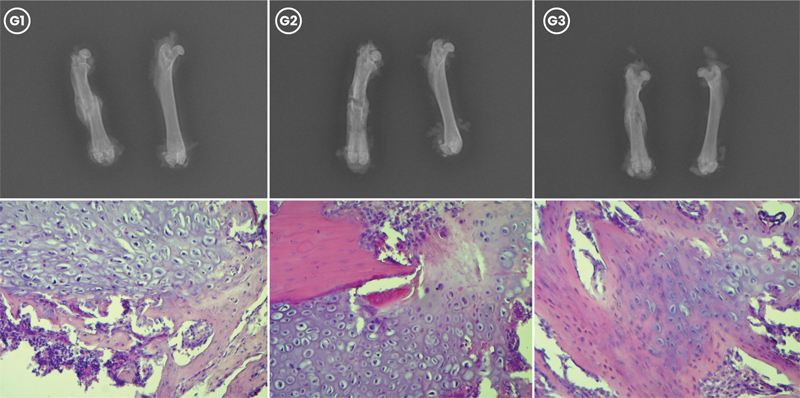
Radiographic and histological evaluation of femoral fracture healing in G1, G2, and G3 at 30 days postfracture. (Top) Radiographs showing consolidated fractures in G1, G2, and G3. (Bottom) Histological sections stained with hematoxylin and eosin, demonstrating bone tissue formation, with G3 showing more advanced callus maturation compared with G1 and G2 (magnification 20x).

The results for the size ratio show the same statistical outcome as the bone callus size, indicating the consistency of the results ( Table 1 ). At 30 days, no significant difference was observed regarding the size and ratio of bone calluses among the groups, as seen in Fig. 2 .

### Histological Evaluation

The analysis of the slides revealed that at 15 days ( Fig. 1 ), all rats presented a predominance of cartilaginous tissue, with no statistical difference between the groups ( Table 2 ). At 30 days ( Fig. 2 ), G3 showed a predominance of bone tissue, while the other groups still had a higher quantity of cartilage ( *p* < 0.05).

**Table 2:** Evaluation of the histological score of bone callus maturation as proposed by Huo et al. 22 at 15 and 30-days

	Day 15	Day 30
**G1**	5.28 ± 0.75 ^a^	7 ± 0.78 ^a^
**G2**	5.5 ± 1.2 ^a^	6.5 ± 0.79 ^a^
**G3**	5.8 ± 1.31 ^a^	7.83 ± 0.71 ^b^

**Abbreviations:** G1, group 1; G2, group 2; G3, group 3. **Notes:** Results are displayed in median ± standard deviation. Different letters in the columns denote significant difference *p* < 0.05.

In the uterine tissue's evaluation, it was observed that there was no difference between G2 and G3 at 15 days, which were significantly larger than G1 in both endometrial and myometrial evaluations. When comparing the groups at 30 days, G3 showed a reduction in endometrial thickness compared with G2. The results of the endometrial and myometrial thickness measurements are shown in Table 3 . Throughout the duration of the experiment, there were no sample losses.

**Table 3:** Evaluation of uterine tissue thickness at 15 and 30-days (in μm)

	Myometrium		Endometrium	
	Day 15	Day 30	Day 15	Day 30
**G1**	382,096 ± 75,017.84 ^a^	298,256 ± 53,430 ^a^	427,311 ± 97,735 ^a^	394,883 ± 79,977 ^a^
**G2**	458,528 ± 105,627.8 ^b^	440,251 ± 58,007 ^b^	783,537 ± 192,171 ^b^	623,729 ± 101,592 ^b^
**G3**	697,511 ± 98,439.72 ^b^	390,452 ± 76,422 ^b^	739,547 ± 131,672 ^b^	510,073 ± 54,705.11 ^c^

**Abbreviations:** G1, group 1; G2, group 2; G3, group. **Notes:** Results are displayed in median ± standard deviation. Different letters in the columns denote significant difference *p* < 0.05.

## Discussion

The oophorectomy performed in this experiment was sufficient to produce an osteoporosis model and consequently alter bone callus formation. Lill et al. 4 indicated that this disease decreases bone callus formation in the initial stages of consolidation and callus mineralization in the final stages, with osteoporotic rats exhibiting a 40% smaller callus than the control group.

Estrogen enhances the osteogenic differentiation of mesenchymal stem cells and osteoblast maturation, favoring bone formation. Additionally, this hormone inhibits osteoclast formation and induces osteoclast apoptosis, limiting bone resorption. Estrogen receptors are highly expressed in osteoblasts and osteocytes, generating protective effects on bone. 23

Estrogen deficiency alters the expression of estrogen target genes, inducing the expression of proinflammatory cytokines such as interleukins (IL)-1, -6, and tumor necrosis factor in the early stages of the consolidation process, reducing osteogenic capacity and delaying callus formation. 24 One of estrogen's actions is to increase TGF-β release, which stimulates collagen and proteoglycan production by mesenchymal cells and osteoblasts, as well as fibronectin production in bone tissue. 25

Beil et al. 7 analyzed the effect of estrogen on fractures in osteoporotic rats and found increased chondrocyte formation in the early stages of the consolidation process in rats treated with estrogen pearls, demonstrating that this hormone stimulates chondral formation. In the present study, the administration of this hormone in the form of nanocapsules induced greater bone callus production at 15 days, as observed in the measurements. Estrogen positively regulates chondrocyte function and maturation, influencing the articular surface and growth plate epiphyses. 25 This observation, supported by Richmond et al., 26 is reinforced by the finding that the bone callus size was significantly larger in the groups treated with it, with G2 and G3 being equal and showing a larger callus than the control group.

It is known that the sooner the inflammatory phase is overcome and the chondral formation process begins, the sooner this tissue will mineralize and the fracture will be repaired. 6 Estrogen's role extends beyond chondrogenesis stimulation in the early stages, as it also influences periosteal consolidation in the final stages of callus formation. Beil et al. 7 found high levels of calcein, a marker of osteoblastic activity, in osteoporotic rats treated with this hormone. Although the present study did not evaluate this marker, we can infer that the macroscopic result found was due to better cellular activity influenced by estrogen.

The greater bone callus formation observed in G2 and G3 showed an acceleration of the process attributed to estrogen, with a statistically better result for the group treated with nanocapsules. The use of nanocapsules improves local action at 15 days, as the group treated with nanocapsules presented a larger bone callus. According to Salimi et al., 27 the use of estrogen in nanoparticle form may allow control of the release rate of the active ingredient, prolonging the pharmacological effect at the site of injury.

At 30 days, radiographic evaluation showed no significant difference in bone callus size among the groups ( *p* > 0.05). Despite G3 having a larger bone callus at 15 days, there were no differences in microscopic callus maturation between groups. Histological evaluation at 30 days revealed better maturation in G3. This improved quality is attributed to the early stimulation of osteochondrogenic cells by estrogen nanocapsules. 28

There was a better bone callus formation in G3 compared with other groups, with a larger callus at 15 days and better quality at 30 days, attributed to improved drug action and permeation. Kaur et al. 29 found higher permeability with nanocapsule formulations in osteoporotic rats, supporting our findings that they significantly enhance drug diffusion to the fracture site compared with conventional methods.

The evaluation of the uterus as a sentinel organ in this study served to monitor the effect of treatments on endometrial tissue. In both treated groups at 15 days, an increase in endometrial and myometrial tissue was observed compared with the untreated group. However, at 30 days, there was a smaller endometrial size in G3 compared with G2. This observation reinforces the results found in the bone callus formation process. Nanoencapsulated drugs reach the action site better, leaving a smaller amount for systemic effects. Silva et al. 30 in their work did not observe systemic effects of topical estrogen treatment, disagreeing with what was found in this study.

### Study Limitations

This study has encountered several limitations. First, we did not assess the minimum effective concentration of estrogen nanocapsules specifically tailored for this application. Furthermore, while our investigation primarily focused on the hormone's local effects on bone healing and its potential systemic implications on uterine tissue, we acknowledge that evaluating other systemic implications beyond uterine tissue could offer a more comprehensive understanding its overall impact. Lastly, we did not explore alternative concentrations in the administration of estrogen, which could reveal dose-dependent effects and provide additional avenues for optimization in future studies.

## Conclusion

Considering the results of this study, estrogen accelerated the fracture consolidation process in osteoporotic rats, mainly by accelerating the chondral phase and culminating in better bone matrix at thirty days. The option of estrogen in nanocapsules obtained a better result than conventional administration. Importantly, the systemic effects, evaluated through uterine tissue analysis, revealed a significant reduction in endometrial thickness in the group treated with nanocapsules compared with those treated with conventional estrogen, indicating fewer systemic side effects.

There seems to be room for the use of local estrogen in nanocapsules for the treatment of postmenopausal osteoporotic fractures. The concentration of this hormone at the fracture site stimulates and accelerates the bone callus formation process, thus avoiding complications inherent to this type of fracture. The administered dose, as well as the reduction of systemic repercussions, should be further analyzed by subsequent studies.
